# Mechanistic Target of Rapamycin Inhibitors in Renal Cell Carcinoma: Potential, Limitations, and Perspectives

**DOI:** 10.3389/fcell.2021.636037

**Published:** 2021-03-15

**Authors:** Seraina Faes, Nicolas Demartines, Olivier Dormond

**Affiliations:** Department of Visceral Surgery, Lausanne University Hospital, University of Lausanne, Lausanne, Switzerland

**Keywords:** mTOR, rapalogs, renal cell carcinoma, HIF-α, angiogenesis

## Abstract

Several elements highlight the importance of the mechanistic target of rapamycin (mTOR) in the biology of renal cell carcinoma (RCC). mTOR signaling pathway is indeed frequently activated in RCC, inducing cancer cell proliferation and survival. In addition, mTOR promotes tumor angiogenesis and regulates the expression of hypoxia-inducible factors that play an important role in a subset of RCC. Despite mTOR protumorigenic effects, mTOR inhibitors have failed to provide long-lasting anticancer benefits in RCC patients, highlighting the need to readdress their role in the treatment of RCC. This review aims to present the rationale and limitations of targeting mTOR in RCC. Future roles of mTOR inhibitors in the treatment of RCC are also discussed, in particular in the context of immunotherapies.

## Introduction

Renal cell carcinoma (RCC), which originates from the kidney epithelium, is the most frequent form of kidney cancer (Hsieh et al., 2017b; Nabi et al., 2018; Kotecha et al., 2019). RCC comprises several histological and molecular subtypes of which clear cell RCC is the most frequent (Moch et al., 2016). Curative surgery is possible in patients with localized RCC (Ljungberg et al., 2015). Unfortunately, many patients present in advanced, metastatic stages at diagnosis, and progression of a localized to an advanced stage is frequent despite surgery. Since advanced RCC is associated with high mortality, a strong need exists to develop appropriate systemic treatments. In this context, major progress has been achieved recently, and today, several therapeutic options exist, including immunotherapy and targeted therapies against vascular endothelial growth factor (VEGF) or mechanistic target of rapamycin (mTOR) signaling pathway (Hsieh et al., 2017b; Kotecha et al., 2019). Nevertheless, the efficacy of these therapies is limited, and disease progression is inevitable in most patients. Therefore, it is important to gain further knowledge regarding the biology of RCC in order to design successful therapies.

mTOR is a serine/threonine kinase that belongs to two distinct protein complexes, named mTORC1 and mTORC2. mTORC1 controls several processes involved in cell growth and proliferation including protein, lipid, and nucleotide synthesis (Saxton and Sabatini, 2017). Inhibition of mTORC1 by allosteric inhibitors generally termed rapalogs results in decreased cell proliferation and accordingly provides anticancer benefits (Waldner et al., 2016; Torii et al., 2020) in different types of cancer, including advanced RCC (Tian et al., 2019). The anticancer effect is however limited, and rapalogs are mostly prescribed as second- or third-line therapies. Although initially viewed as promising, mTORC1 inhibitors did not meet the expectations in RCC, and their roles need therefore to be readdressed. Here, the biologic rationale and limitations to use mTOR inhibitors in advanced RCC are reviewed. In addition, future roles that mTOR inhibitors might endorse in the treatment of advanced RCC are discussed.

## Activation of Mechanistic Target of Rapamycin in Renal Cell Carcinoma

**Figure 1:**
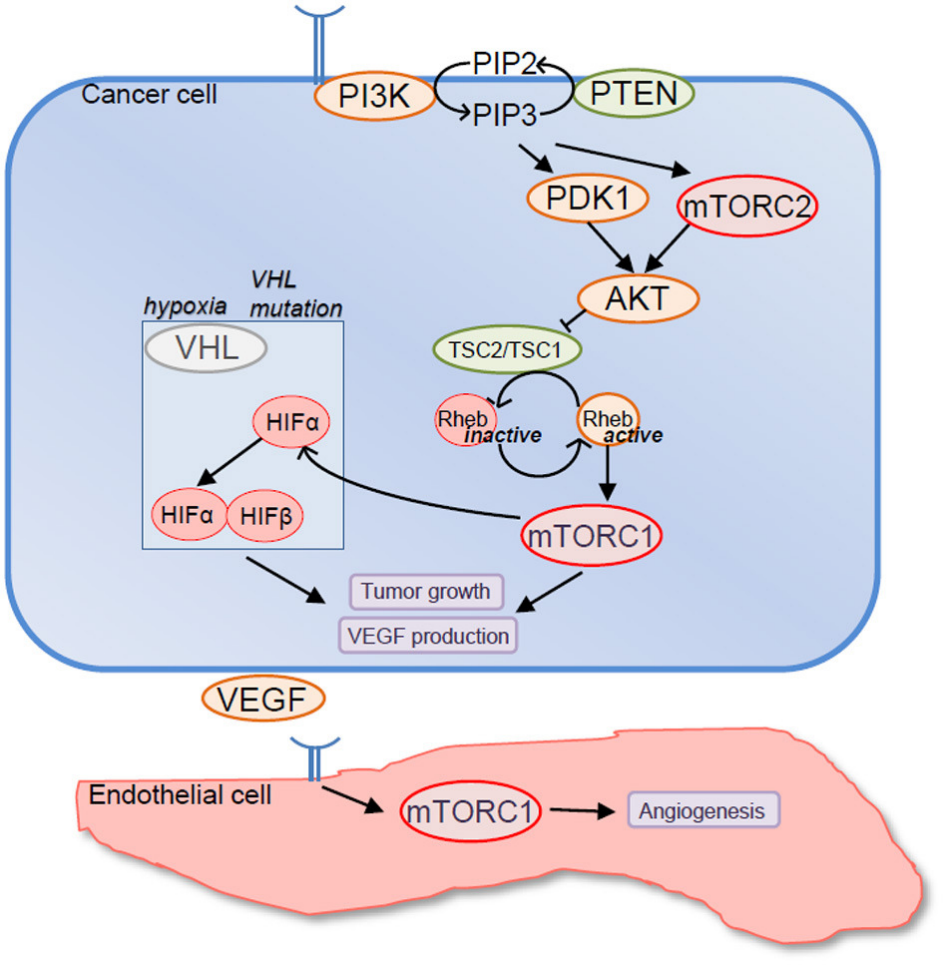
Rationale to target mechanistic target of rapamycin (mTOR) in renal cell carcinoma (RCC). Components of mTOR signaling pathway are frequently mutated in RCC resulting in mTORC1 overactivation. In turn, mTORC1 promotes tumor cell growth and VEGF production either directly or by upregulating expression levels of hypoxia-inducible factor (HIF)-α factors. In addition mTORC1 promotes endothelial cell functions that are relevant to tumor angiogenesis including endothelial cell proliferation, survival, and migration.

A major reason to target mTOR in RCC relies on the observation that, overall, mTOR contributes to cancer cell proliferation and survival, and mTOR signaling pathway is often activated in advanced RCC (Figure 1). In fact, genetic alterations of components of mTOR signaling pathway are frequently observed in RCC, underscoring the possible importance of mTOR in RCC development. For instance, 28% of clear cell RCC presents activating mutations of PI3K/AKT/mTOR signaling pathway that correlate with worse outcome (Cancer Genome Atlas Research, 2013). Similarly, 23% of chromophobe RCC displays genetic modifications of mTOR signaling pathway (Davis et al., 2014). Furthermore, expression of the activated forms of various components of the PI3K/AKT/mTOR signaling pathway was detected in a high percentage of RCC by immunohistochemistry (Lin et al., 2006; Pantuck et al., 2007; Abou Youssif et al., 2011).

mTOR inhibitors exert their antitumor effects in part by reducing cancer cell proliferation via blocking the G1-S cell cycle transition (Dufour et al., 2011). Decreased expression of cyclin D1 and upregulated levels of p27^Kip1^ have been observed in cancer cells as molecular basis for cell cycle blockage. Consistent with these findings, mTOR inhibitors induce G1 block in RCC cell lines *in vitro* and reduce cancer cell proliferation in RCC xenografts (Luan et al., 2003; Zhang et al., 2013; Zheng et al., 2015). Despite promising preclinical experiments, mTOR targeting agents have been less successful than expected in RCC patients. An initial phase III study tested the rapalog temsirolimus against interferon-α among patients with advanced RCC and poor prognosis (Hudes et al., 2007). Median overall survival was longer in the temsirolimus group compared with the interferon-α group (10.9 vs. 7.3 months). Similarly, everolimus was reported to increase progression-free survival compared with placebo in advanced RCC patients that had progressed on multi-targeted tyrosine kinase inhibitors (Motzer et al., 2008). Therefore, despite extensive activation of the PI3K/AKT/mTOR signaling pathway in advanced RCC, rapalogs display modest anticancer activity, suggesting that tumors do not depend on mTORC1 for growth and survival. This could be explained by the fact that upstream activators of mTORC1 such as PI3K or AKT activate several other downstream effectors that possess protumorigenic effects besides mTORC1 (Faes and Dormond, 2015). In addition, several resistance mechanisms might explain the limited efficacy of rapalogs including rapalog-resistant mutations of mTOR, activation of alternate proliferative pathways following mTORC1 inhibition, or tumor heterogeneity (Faes et al., 2017a). Finally, rapalogs only partially inhibit mTORC1, and therefore, a complete mTORC1 inhibition might be necessary to achieve better clinical outcome (Thoreen et al., 2009). In this context, kinase inhibitors of mTOR that display a complete inhibition of mTORC1 and that in contrast to rapalogs also inhibit mTORC2 have been developed (Benjamin et al., 2011). Preclinical studies have demonstrated that kinase inhibitor of mTOR are superior to rapalogs in RCC models (Cho et al., 2010; Ingels et al., 2014; Zheng et al., 2015). Unfortunately, such results were not confirmed in clinical settings. Two randomized phase 2 trials showed that the kinase inhibitor of mTOR AZD2014 (Powles et al., 2016b) and the dual PI3K/mTOR inhibitor GDC-0980 (Powles et al., 2016a) were inferior to everolimus in RCC patients who had progressed following exposure to VEGF pathway targeting therapies. Finally, a third-generation inhibitor named rapalink-1 and composed of rapamycin linked to the kinase inhibitor of mTOR MLN0128 provides improved anticancer efficacy in RCC models compared with temsirolimus (Kuroshima et al., 2020).

Nevertheless, a small minority of RCC patients respond to rapalogs, highlighting the need to identify biomarkers that could predict patients benefiting from rapalogs. Conceptually, cancers in which mTOR is a driving force with few heterogeneity represent the ideal candidate (Rodriguez-Moreno et al., 2017). In this context, the histologic subtype is not helpful in selecting patients. It was indeed reported that temsirolimus was efficient in patients regardless of tumor histology (Dutcher et al., 2009). Similar results were observed in a retrospective study analyzing the effect of temsirolimus and everolimus in RCC with sarcomatoid or non-clear cell histologies (Voss et al., 2014a). Genetic analysis of mTOR pathway mutations has also provided mitigated results so far (Table 1). In fact, two out of five patients who showed exceptional response to rapalogs lacked mTOR signaling pathway activating mutations (Voss et al., 2014b). Similarly, in another cohort of RCC patients treated with rapalogs, 56% of responders had no genetic finding to explain their response (Kwiatkowski et al., 2016). Epigenetic mechanisms or direct effects of rapalogs on the tumor microenvironment might be at play. Nevertheless, mutations in tuberous sclerosis proteins *TSC1* or *TSC2*, close upstream regulators of mTORC1, and *mTOR* are more common among rapalog responders (Kwiatkowski et al., 2016; Roldan-Romero et al., 2017). More promisingly, loss of PTEN expression, and not loss-of-function mutations, has recently been associated with everolimus therapeutic response (Voss et al., 2019; Roldan-Romero et al., 2020). Clearly, additional work is however required to identify reliable and robust combinations of biomarkers of response that can be used in clinic. Interestingly, a complete analysis of exceptional responders to rapalogs (mean progression-free survival of 28 months) revealed convergent mutations resulting in mTOR pathway activation (Voss et al., 2014b). It was therefore proposed that RCC development behaves rather like a braided river than a branching tree (Hsieh and Cheng, 2016; Hsieh et al., 2017a). This parallel convergent evolution of kidney cancer would thus offer significant therapeutic opportunities despite tumor heterogeneity.

**Table 1:** RCC genetic analysis and response to rapalogs.

**Patients**	**Genetic alterations**	**Results**		**References**
79 selected RCC patients treated with rapalogs	**Mutation** *TSC1* or *TSC2* or *mTOR* No mTOR pathway mutation	Responders^a^ 28% 56%	Non-responders^b^ 11% 78%	Kwiatkowski et al., 2016
45 RCC patients treated with rapalogs (five harboring mTOR related mutations)	**Mutation** *mTOR* early event *mTOR* *mTOR* *TSC1* *TSC2*	Response PR SD SD PR PD	PFS (months) 89 9 3 11	Roldan-Romero et al., 2017
Exceptional responder	**Mutation** *mTOR* early event	Disease free after 8 years' temsirolimus treatment		Rodriguez-Moreno et al., 2017
184 everolimus treated RCC patients	**IHC staining** PTEN negative PTEN positive	PFS (months) 10.5 5.3 No correlation with *TSC1, TSC2* or *mTOR* mutations		Voss et al., 2019
105 rapalogs treated RCC patients	**IHC staining** PTEN negative PTEN positive	Responders^c^ 48% 18%	Non-responders^d^ 52% 82%	Roldan-Romero et al., 2020

*PR, partial response; SD, stable disease; PD, progressive disease; PFS, progression-free survival; IHC, immunohistochemistry; RCC, renal cell carcinoma*.

a *Responders: PR or SD with any tumor shrinkage for 6 months*.

b *Non-responders: PD during the first 3 months of therapy*.

c *Responders: PR or SD and at least 6 months PFS*.

d *Non-responders: PD or SD of <6 months PFS*.

Since rapalogs alone provide little benefits, several trials have explored mTOR inhibitors in combination therapies. In particular, combinations of rapalogs with anti-angiogenic drugs including, sorafenib, sunitinib, and bevacizumab have been tested (Ravaud et al., 2013). Most trials were however discontinued or required dose modification due to drug toxicity. In fact mTOR inhibitors are associated with substantial side effects such as mucositis, rash, myelosuppression, hyperglycemia, hypophosphatemia, hypercholesterolemia, and pneumonitis, limiting their application in cancer patients (Rodriguez-Pascual et al., 2010; Pallet and Legendre, 2013). Nevertheless, combining lenvatinib with everolimus resulted in an acceptable safety profile and has been approved for RCC patients who had received prior antiangiogenic treatments (Motzer et al., 2015).

## Anti-angiogenic Effects of Mechanistic Target of Rapamycin Inhibitors in Renal Cell Carcinoma

RCCs are highly vascularized tumors, and the therapeutic benefits of anti-VEGF signaling therapies underline the role of angiogenesis in RCC development (Hsieh et al., 2017b). In this context, targeting mTOR represents a treatment strategy, as mTOR controls several processes implicated in tumor angiogenesis (Faes et al., 2017b). In fact, mTOR is an important signaling intermediary that regulates endothelial functions relevant to angiogenesis such as proliferation, survival, and migration (Akselband et al., 1991; Bruns et al., 2004; Dormond et al., 2007). Furthermore, mTOR modulates tumor angiogenesis by regulating the production of pro-angiogenic factors in particular VEGF (Guba et al., 2002). Accordingly, mTOR inhibitors decrease tumor angiogenesis in a variety of preclinical models (Faes et al., 2017b). However, the contribution of the anti-angiogenic effects of rapalogs in RCC patients remains to be demonstrated. Of note, combining the anti-VEGF bevacizumab to temsirolimus did not provide better results than bevacizumab with interferon-α, suggesting that bevacizumab and temsirolimus share inhibition of angiogenesis as a common anticancer effect (Rini et al., 2014). Nevertheless, the limited efficacy of rapalogs in advanced RCC challenges the anti-angiogenic efficacy of mTOR inhibitors. In fact, mTOR inhibitors had no impact on microvessel density of RCC xenografts, suggesting that in certain circumstances, tumor blood vessels are not sensitive to mTOR inhibitors, or the anti-angiogenic effects might only be transient (Cho et al., 2010; Ellis et al., 2012).

## Regulation of Hypoxia-Inducible Factors by Mechanistic Target of Rapamycin

Clear cell RCC frequently harbors loss-of-function mutations of the tumor suppressor gene *Von Hippel–Lindau* (*VHL*). Consequently, hypoxia-inducible factors (HIF-1α and HIF-2α) accumulate, leading to a constant hypoxic tumor response that promotes tumor growth and angiogenesis despite the presence of oxygen (Patel et al., 2006; Shen and Kaelin, 2013). Therefore, targeting HIFs might influence RCC progression (Schodel et al., 2016). Interestingly, in contrast to initial thoughts, emerging evidence has now demonstrated a divergent role for HIF-α factors in RCC biology where HIF-1α reduces and HIF-2α promotes RCC growth (Kondo et al., 2002, 2003; Shen et al., 2011; Gudas et al., 2014; Hoefflin et al., 2020).

Several reports have evidenced that mTOR decreases expression of HIF-α factors and therefore might influence RCC growth (Faes et al., 2017b). Consistent with this hypothesis, it was demonstrated that *VHL* mutation determines RCC sensitivity to temsirolimus in a mouse model of RCC (Thomas et al., 2006). Rapalogs decrease HIF-1α expression via different mechanisms including HIF-1α mRNA transcription, mRNA translation, protein stabilization, and transcriptional activity (Faes et al., 2017b). In contrast, HIF-2α expression depends on mTORC2 activity and is accordingly not affected by rapalogs (Toschi et al., 2008). In the context of RCC, opposing results have been reported regarding the effect of mTOR inhibitors on HIF-α factor expression. It was shown that temsirolimus reduces both HIF-1α and HIF-2α expression in RCC *in vitro* (Thomas et al., 2006). In contrast, whereas the dual PI3K/mTOR inhibitor NVP-BEZ235 decreased HIF-2α expression in 786-0, A498, Caki-1, and Caki-2 RCC cell lines, rapamycin had no significant effects (Cho et al., 2010). Taken together, these studies highlight the complex interrelationship between mTOR and HIF-α factors and suggest that preferential inhibition of HIF-1α expression over HIF-2α by rapalogs might provide detrimental protumorigenic signals. Nevertheless, a complete understanding of the role of HIF-α factors in RCC and the consequences of mTOR inhibition on their activities is necessary.

## Future Direction: Combining Mechanistic Target of Rapamycin Inhibitors With Immunotherapies

Following major therapeutic success by immunotherapy in melanoma, lot of efforts are deployed to design immunotherapy-based protocols in RCC. Interleukin-2 and interferon-α were initially used in patients with advanced RCC, suggesting that RCC might be particularly sensitive to immunotherapies. Accordingly, numerous clinical trials are currently exploring the effects of immunotherapy alone or in combination with various targeted therapies (Garje et al., 2020). Since rapalogs are mainly used to prevent rejection of transplanted organs, their use with immunotherapies seems aberrant at first look. However, emerging studies have demonstrated that mTOR inhibitors display immunostimulatory effects. Rapamycin increases memory CD8^+^ T cell differentiation following viral infection (Araki et al., 2009). Rapamycin treatment also induces stem-cell like memory T cells during activation of human naïve T cells (Scholz et al., 2016). In cancer, preclinical studies have shown that rapalogs enhance the tumor response to different types of immunotherapies including vaccines, adoptive T cell therapy, and checkpoint inhibitors (Thomas et al., 2011; Wang et al., 2011; Amiel et al., 2012; Li et al., 2012; Diken et al., 2013; Mineharu et al., 2014; Moore et al., 2016). Therefore, additional studies are needed to fully characterize the conditions in which mTOR inhibition results in immune stimulation or inhibition. Interestingly, the immune modulatory properties of rapalogs were assessed in RCC patients, and results confirmed that mTOR inhibitors provide opposing effects on the antitumor immune response (Beziaud et al., 2016; Huijts et al., 2017). In most patients, the rapalog everolimus promoted expansion of ${\text{FoxP}}_{3}^{+}$ regulatory T cells (T_regs_) and increased spontaneous tumor-specific TH_1_ response. Importantly, in a subset of patients, everolimus decreased T_regs_ levels while increasing TH_1_ response, which was associated with a longer progression-free survival. This suggests that the antitumor effects of rapalogs occur in part via modulation of the antitumor immune response and provide an additional rationale to combine mTOR inhibitors with immunotherapies.

The endothelial barrier is an important obstacle to recognize when considering combining anticancer agents with immunotherapies (Schmittnaegel and De Palma, 2017; Uldry et al., 2017). In fact, tumor blood perfusion is frequently reduced due to abnormal blood vessels, resulting in hypoxia and decreased delivery of anticancer agents and immune cells to tumors (Martin et al., 2019). Accordingly, tumor blood vessel normalization with anti-angiogenic drugs improves cancer immunotherapy by in part augmenting T cell extravasation (Allen et al., 2017; Schmittnaegel et al., 2017; Mpekris et al., 2020). Preclinical studies demonstrated contrasting results regarding the effects of mTOR inhibitors on tumor blood vessel normalization. On the one hand, reduction of vessel permeability and increased tumor perfusion by mTOR inhibitors were observed in different cancer models (Schnell et al., 2008; Zhang et al., 2011; Myers et al., 2012). On the other hand, absence of effects was also noted (Lane et al., 2009; Ellis et al., 2012). Therefore, applying mTOR inhibitors in conditions where they induce vessel normalization might be particularly beneficial with immunotherapies. Tumor blood vessels also actively participate to the recruitment of leukocytes into tumors by expressing adhesion molecules such as intercellular adhesion molecule-1 (ICAM-1) or vascular cell adhesion molecule-1 (VCAM-1). In addition, they modulate T cell activity by expressing MHC class I and class II as well as co-stimulatory and co-inhibitory molecules (Choi et al., 2004). Of note, it was reported that mTOR inhibitors upregulate PD-L1 and PD-L2 and reduce VCAM-1 expression on endothelial cells (Wang et al., 2013, 2014). Consequently, rapamycin pretreatment of human arterial allografts decreased infiltration of artery intima by effector T cells. Although these findings need to be investigated in tumor models, they suggest that mTOR inhibitors might reinforce the tumor endothelial barrier and therefore counteract their benefits in the context of immunotherapy.

## Conclusions

Despite clear implications of mTOR signaling pathway in RCC development and progression, inhibition of mTOR through rapalogs did not provide major and long-lasting anticancer benefits in patients. Whereas, mTOR is frequently activated in RCC and participates in tumor growth, RCC harbors major genetic heterogeneity, implying that many driving forces, not limited to mTOR, participate in tumor growth. Combination therapies might therefore provide additional antitumor effects, albeit increased toxicity. Although several preclinical studies have demonstrated that mTOR inhibitors decrease tumor angiogenesis, this specific mechanism in the context of RCC has not been thoroughly investigated. More importantly, some investigations in RCC mouse models did not find any inhibitory effect of rapalogs on the tumor vasculature. Finally, in a subset of RCC that presents *VHL* mutation, the preferential inhibition of HIF-1α over HIF-2α by rapalogs might decrease the tumor-suppressing effects of HIF-1α, counteracting the anticancer efficacy of rapalogs. Given the success of immunotherapies, future investigations addressing the role of mTOR inhibitors in RCC will certainly focus on their immunostimulatory effects. Accordingly, dissecting the conditions where mTOR inhibitors exert immunostimulatory instead of immunosuppressing activities will be key. Interestingly, a study demonstrated that some RCC patients preferentially presented increased antitumor response under rapalog treatment, highlighting their therapeutic potential in combination with immunotherapy.

## Author Contributions

SF and OD designed the manuscript. SF drafted the manuscript. OD and ND revised the manuscript. All authors have approved the final version of the manuscript.

## Conflict of Interest

The authors declare that the research was conducted in the absence of any commercial or financial relationships that could be construed as a potential conflict of interest.
